# Nontargeted metabolomics reveals the potential mechanism underlying the association between birthweight and metabolic disturbances

**DOI:** 10.1186/s12884-023-05346-6

**Published:** 2023-01-09

**Authors:** Xiao Zhai, Jieying Liu, Miao Yu, Qian Zhang, Ming Li, Nan Zhao, Juntao Liu, Yingna Song, Liangkun Ma, Rongrong Li, Zongxu Qiao, Guifen Zhao, Ruiping Wang, Xinhua Xiao

**Affiliations:** 1 Department of Endocrinology, Key Laboratory of Endocrinology, Ministry of Health, Peking Union Medical College Hospital, Chinese Academy of Medical Sciences & Peking Union Medical College, Beijing, 100730 China; 2 Department of Medical Research Center, Peking Union Medical College Hospital, Chinese Academy of Medical Sciences & Peking Union Medical College, Beijing, 100730 China; 3 Department of Obstetrics & Gynecology, Peking Union Medical College Hospital, Chinese Academy of Medical Sciences & Peking Union Medical College, Beijing, 100730 China; 4 Department of Clinical Nutrition, Peking Union Medical College Hospital, Chinese Academy of Medical Sciences & Peking Union Medical College, Beijing, 100730 China; 5 Department of Obstetrics & Gynecology, Xingtai People’s Hospital, Xingtai, Hebei 054000 People’s Republic of China

**Keywords:** Metabolomics, Newborn birthweight, Metabolic disturbances

## Abstract

**Aims:**

The aim of this study was to characterize the metabolites associated with small- and large-gestational-age newborns in maternal and cord blood, and to investigate potential mechanisms underlying the association between birthweight and metabolic disturbances.

**Research design and methods:**

We recorded detailed anthropometric data of mother-offspring dyads. Untargeted metabolomic assays were performed on 67 pairs of cord blood and maternal fasting plasma samples including 16 pairs of small-for-gestational (SGA, < 10th percentile) dyads, 28 pairs of appropriate-for-gestational (AGA, approximate 50 percentile) dyads, and 23 pairs of large-for-gestational (LGA, > 90th percentile) dyads. The association of metabolites with newborn birthweight was conducted to screen for metabolites with U-shaped and line-shaped distributions. The association of metabolites with maternal and fetal phenotypes was also performed.

**Results:**

We found 2 types of metabolites that changed in different patterns according to newborn birthweight. One type of metabolite exhibited a “U-shaped” trend of abundance fluctuation in the SGA-AGA-LGA groups. The results demonstrated that cuminaldehyde level was lower in the SGA and LGA groups, and its abundance in cord blood was negatively correlated with maternal BMI (*r* = -0.352 *p* = 0.009) and weight gain (*r* = -0.267 *p* = 0.043). 2-Methoxy-estradiol-17b 3-glucuronide, which showed enrichment in the SGA and LGA groups, was positively correlated with homocysteine (*r* = 0.44, *p* < 0.001) and free fatty acid (*r* = 0.42, *p* < 0.001) in maternal blood. Serotonin and 13(S)-HODE were the second type of metabolites, denoted as “line-shaped”, which both showed increasing trends in the SGA-AGA-LGA groups in both maternal and cord blood and were both significantly positively correlated with maternal BMI before pregnancy. Moreover, cuminaldehyde, serotonin, 13(S)-HODE and some lipid metabolites showed a strong correlation between maternal and cord blood.

**Conclusions:**

These investigations demonstrate broad-scale metabolomic differences associated with newborn birthweight in both pregnant women and their newborns. The U-shaped metabolites associated with both the SGA and LGA groups might explain the U-shaped association between birthweight and metabolic dysregulation. The line-shaped metabolites might participate in intrauterine growth regulation. These observations might help to provide new insights into the insulin resistance and the risk of metabolic disturbance of SGA and LGA babies in adulthood and might identify potential new markers for adverse newborn outcomes in pregnant women.

**Supplementary Information:**

The online version contains supplementary material available at 10.1186/s12884-023-05346-6.

## Introduction

Disturbances in fetal growth could greatly increase the susceptibility to chronic metabolic diseases in adulthood, mainly obesity, insulin resistance, impaired glucose tolerance, dyslipidemia, and cardiovascular diseases [1–3]. It has been recognized for years that small-for-gestational-age (SGA) infants are associated with a high risk of developing metabolic dysregulations in adulthood [3–7]. In addition, infants born as large-for-gestational-age (LGA) are also at a higher risk of T2DM and obesity [8]. This demonstrates the U-shaped relationship between birthweight and the risk of T2DM [9].The hypothesis of “fetal origins of adult diseases” proposed by Barker et al*.* indicates that intrauterine factors have long-term programming effects on fetal development and lead to increased vulnerability to chronic diseases later in life, often in adulthood [1]. This concept was initially supported by studies on children with SGA [10] and maternal malnutrition [11] associated with increased susceptibility to chronic diseases later in life. Studies on prenatal famine during the Dutch Hunger Winter [12] and adults born during the Chinese famine between 1959 and 1961 [13] found that individuals who were exposed to famine in utero were more prone to be overweight and have T2DM. The thrifty phenotype attributes the relationship of adverse intrauterine growth and subsequent increased metabolic risk to the compensatory response to undernutrition status in early life, leading to permanent changes in metabolism [1].

While the foundational studies of this “fetal origins of adult diseases” theory focused on the influence of prenatal undernutrition on offspring, an increasing body of studies have focused on the impact of maternal obesity and diabetes during pregnancy on offspring. Evidence has shown that maternal obesity and overnutrition are linked to a higher incidence of macrosomia or LGA and have a potential impact on future metabolic risk [2, 14, 15]. For instance, maternal prepregnancy obesity and excessive gestational weight gain are associated with higher offspring birthweight and childhood adiposity [16]. Moreover, a positive association has been discovered between maternal diabetes or third-trimester glucose tolerance with higher offspring birth weight and youth-onset T2DM incidence [17].

However, the mechanism underlying the association between high or low birthweight and T2DM risk remains unclear. It is quite interesting that high- and low-birth-weight infants are likely exposed to different intrauterine environments; yet, they both have an elevated tendency to develop metabolic diseases in adulthood. Therefore, we hypothesized that high- and low-birthweight infants could share similar metabolic alterations reflecting underlying important biomolecular mechanisms linked to these processes.

There is an urgent need to understand the complex intrauterine biomolecular perturbations and to identify individuals at risk of metabolic disturbances. Since the exchange of nutrients for fetal metabolism by the placenta is essential for fetal growth and due to the placental barrier, most substances that pass through the barrier are small and hydrophobic molecules, such as glucose, amino acids and fatty acids [18]. The analysis of the metabolite profile in maternal blood and fetal cord blood might reflect parts of the material exchange and depict the intrauterine environment at a glance. Consequently, using rapidly developing metabolomics technologies that focus on the quantity of low molecular weight (< 1500 Da) metabolites offers an integrative perspective into this maternal–fetal metabolism status. Full-scan nontargeted Q-TOF coupled with liquid chromatography can provide excellent robustness, and hundreds of samples can be profiled [19].

In this study, we aimed to characterize the metabolic phenotypes of pregnant women and their newborn babies with high or low birth weight, identify metabolic perturbations linked to birth weight, and explore potential biomolecular mechanisms linked to abnormal birth weight.

## Research design and methods

### Data and sample collection

This study recruited mother–offspring dyads from the outpatient clinic and/or the delivery ward of the Department of Obstetrics and Gynecology at Peking Union Medical College Hospital (PUMCH) and Xingtai People's Hospital (XTPH) in China from September 2017 to July 2018. The inclusion criteria were as follows: being singleton pregnant, maternal age between 18 and 45, and gestational age (GA) between 37 and 42 weeks. Participants who had smoked or had alcohol use during pregnancy and those who had been diagnosed with hypertension or preeclampsia were excluded from this study. The study protocol was approved by the ethics committees of PUMCH and the ethics committee of XTRH, and written informed consent was obtained from all participants prior to recruitment.

A total of 165 mother–offspring dyads were admitted to our study. According to the birth weight of neonates and the Chinese neonatal birth weight report [20], the birth weight of neonates are classified as SGA (< 10th percentile), appropriate for gestational (AGA) (approximately 50th percentile), and LGA (> 90th percentile) based on gestational age.16 pairs of SGA dyads, 28 pairs of AGA dyads, and 23 pairs of LGA dyads were selected for this study. Participating mothers underwent a 75-g oral glucose tolerance test (OGTT) between 24 and 28 weeks of gestation. Maternal anthropometric measurements, including height, weight, and mean arterial pressure, were measured by doctors. Information on maternal age, parity, past medical history, prepregnancy weight, and gestational weight gain were collected from their pregnancy health records. Prepregnancy body mass index (BMI) was calculated as prepregnancy weight in kilograms divided by measured height in meters squared (kg/m^2^). Birth weight, newborn sex, mode of delivery, and gestational age were obtained from the hospital delivery records.

Maternal blood samples were collected in a fasting state between 37 and 42 weeks gestational age during routine blood sampling from the outpatient clinic. Cord vein blood samples were obtained within 10 min of delivery. The blood samples were collected into 2-ml EDTA containers and placed on ice. The blood was immediately centrifuged (3000 rpm, 15 min) at 4 °C, plasma was separated, and aliquots (0.2 ml) were rapidly stored at -80 °C until metabolomic assays.

### Conventional metabolite analysis

Conventional metabolites were measured on a Beckman Coulter AU5800. Hypersensitive C-reactive protein (hs-CRP), C-peptide (C-Pep) and insulin were measured using reagents from Beckman (Brea, CA). Glucose, triglycerides (TGs), total cholesterol (TC), high-density lipoprotein cholesterol (HDL-C), low-density lipoprotein cholesterol (LDL-C), lipoprotein(a) (Lp(a)) and free fatty acids (FFAs) were measured using reagents from Sekisui (Tokyo, JPN). Additionally, homocysteine (HCY) was measured using reagents from Leadman (Beijing, CHN), and glycated albumin (GA) was measured using reagents from Asahi (Tokyo, JPN). Leptin and adiponectin were analyzed by an enzyme-linked immunosorbent assay (Crystal Chemistry, IL, USA).

### Nontargeted metabolomics assays

The nontargeted metabolomics assays were conducted as previously reported [19]. Specifically, a system coupling ultraperformance liquid chromatography (UPLC, Waters ACQUITY UPLC I-Class) to time-of-flight (TOF, Waters XevoG2-XS Qtof) mass spectrometry was used to analyze the full range of metabolites present in plasma. Methanol at a ratio of 3:1 (vol/vol) to the sample was added for protein removal overnight at -20 °C. The samples were centrifuged, and the supernatants were analyzed directly. Quality control (QC) pools were constructed using equal volumes from all the samples, prepared for analysis as described above, and injected every 10 samples for all the runs. The mass spectrometers (MSs) adopted both positive and negative ionization modes, which helped detect more compounds. Progenesis QI software (Waters) performs peak picking and drift alignment (retention time and accurate mass) based on QC samples [17]. The annotation of metabolites was in Progenesis QI, the spectra are matched against reference spectra in HMDB (http://www.hmdb.ca/), METLIN and KEGG databases [21].

### Statistical analyses

#### Conventional metabolites and group comparisons

All statistical analyses were performed in SPSS version 25.0 (SPSS, Chicago, IL). Means and SDs of conventional metabolites were calculated. Categorical variable frequencies and continuous variable means were compared between 3 groups using Fisher’s exact tests and one-way ANOVA, respectively.

#### Nontargeted metabolomic analysis

As previously reported [22], multivariate analysis was conducted using partial least squares regression discriminant analysis (PLS-DA) in SGA/AGA/LGA group comparisons in SIMCA version 14.1 (MKS Umetrics AB, Umea, Sweden). Orthogonal partial least squares regression discriminant analysis (OPLS-DA) was used in SGA/AGA and AGA/LGA maternal and fetal comparisons. Three parameters, including R^2^ and Q^2^, were used to evaluate the quality and reliability of these models. Individual metabolites were selected based on the variable importance in projection (VIP), and metabolites in which VIP > 1 were selected and processed for the following meta-analysis.

The peak intensity data, which represent the metabolite abundance in each sample, were normalized using Z score transformation for meta-analysis. We first identified the overlapping metabolites between the maternal and fetal groups in a Venn diagram using the R package VennDiagram and selected the metabolites with the same change direction presented in the heatmap using the R package ComplexHeatmap. In this study, 2 types of metabolites were separately analyzed, including U-shaped (higher or lower in both the SGA/LGA group) and line-shaped (the abundance in the SGA/AGA/LGA group trend according to the birthweight of offspring). MetaboAnalyst 5.0 (https://www.metaboanalyst.ca/) was used for metabolomic pathway analysis [23].

#### Statistical-analysis

Continuous variables were described as the means with SD. Categorical variables are represented as frequencies with proportions. The correlation of the maternal and fetal metabolome was calculated and drawn using the Spearman method by the R package “ggplot2”. Spearman’s correlation analysis was used to assay the correlation between metabolites and clinical parameters, as for model 1. For model 2, Partial correlation coefficients were calculated to evaluate the association between metabolites and clinical parameters after adjusting for confounders. r coefficient was calculated after adjusting for maternal age, parity and gestational age, and fetal sex. A P value < 0.05 (two-sided) was considered statistically significant.

## Results

### Population characteristics

The following 67 pairs of mother–offspring dyads were included in this study: 16 pairs of SGA (< 10th percentile) dyads, 28 pairs of AGA dyads (approximately 50th percentile), and 23 pairs of LGA (> 90th percentile) (Table 1). Most of the clinical characteristics were similar among the 3 groups. Maternal age, gestational age, maternal GDM proportion and most clinical biochemical indices, including fasting plasma glucose, insulin, FFA, TC, TG, leptin and adiponectin, showed no significant difference between SGA, AGA and LGA mothers. The prepregnancy BMIs were 22.4, 23.4 and 25.1 in the SGA, AGA and LGA groups, respectively (*p* = 0.03). Moreover, HDL-C in LGA mothers (Supplemental Table S1) was significantly lower than that in the other two groups (*p* = 0.01). Regarding newborn characteristics, cord blood insulin (*p* = 0.04) and leptin (*p* = 0.02) were significantly higher in the LGA group.

**Table 1:** Demographics of mothers and their offspring

Characteristic	SGA	AGA	LGA	*P*
***N***	16	28	23	
**Field center, *N* (%)**				
PUMCH, Beijing	6 (37.5)	11 (39.3)	7 (30.4)	0.81
XTPH, Hebei	10 (62.5)	17 (60.7)	16 (69.6)	
**Maternal parity, *N* (%)**				
First child	11 (68.8)	8 (28.6)	14 (60.9)	**0.02**
Subsequent child	5 (31.2)	20 (71.4)	9 (39.1)	
**New born sex, *N* (%)**				
Male	12 (75.0)	15 (53.6)	14 (60.9)	0.41
Female	4 (25.0)	13 (46.4)	9 (39.1)	
**Mode of delivery, *N* (%)**				
Vaginal	7 (43.8)	10 (35.7)	3 (13.0)	0.08
Cesarean section	9 (56.2)	18 (64.3)	20 (87.0)	
**Maternal Characteristics, Mean (SD)**				
Maternal age (years)	31.4 (4.6)	33.7 (6.7)	30.9 (5.3)	0.20
Gestational age (weeks)	38.7 (1.1)	38.4 (0.9)	39.0 (1.1)	0.13
Prepregnancy BMI (kg/m^2^)	22.4 (3.4)	23.4 (3.5)	25.1 (2.6)	**0.03**
Gestational weight gain(kg)	14.8 (5.2)	14.2 (6.0)	15.5 (5.8)	0.74
Mean arterial pressure(mmHg)	89.5 (10.2)	89.2 (9.2)	89.6 (9.7)	0.94
Maternal GDM, *N* (%)	8 (50.0)	18 (64.3)	13 (56.5)	0.67
Fasting plasma glucose(mmol/L)	4.6 (0.7)	4.7 (0.6)	4.6 (0.5)	0.78
Fasting C-peptide (ng/mL)	2.7 (2.3)	2.2 (0.6)	2.3 (0.7)	0.37
Fasting Insulin (µIU/mL)	10.7 (6.8)	11.8 (3.8)	13.7 (5.6)	0.27
Leptin (ng/mL)	644.9 (494.1)	413.5 (311.1)	518.5 (391.8)	0.17
Adiponectin (ng/mL)	6135.8 (3230.1)	6908.0 (5258.9)	6641.8 (393.2)	0.88
**Newborn Characteristics, Mean (SD)**				
Birthweight(g)	2739.4 (250.7)	3300.4 (133.2)	4097.0 (238.2)	** < 0.001**
Cord glucose(mmol/L)	4.4 (1.3)	4.5 (1.6)	4.4 (2.1)	0.98
Cord C-peptide (ng/mL)	0.6 (0.2)	1.1 (1.3)	1.4 (0.9)	0.08
Cord Insulin (µIU/mL)	4.2 (2.2)	6.9 (3.9)	14.9 (21.1)	**0.04**
Cord Leptin (ng/mL)	43.7 (42.8)	118.7 (203.5)	355.6 (531.5)	**0.02**
Cord Adiponectin (ng/mL)	26,221.2 (8062.6)	27,014.2(10,613.8)	28,995.6 (9760.0)	0.72

Data are n (%) or means?±?SD unless otherwise indicated. Categorical variable frequencies and continuous variable means were compared for SGA/AGA/LGA mothers using Fisher’s exact tests and one-way ANOVA, respectively

### Metabolomic analysis

**Fig. 1:**
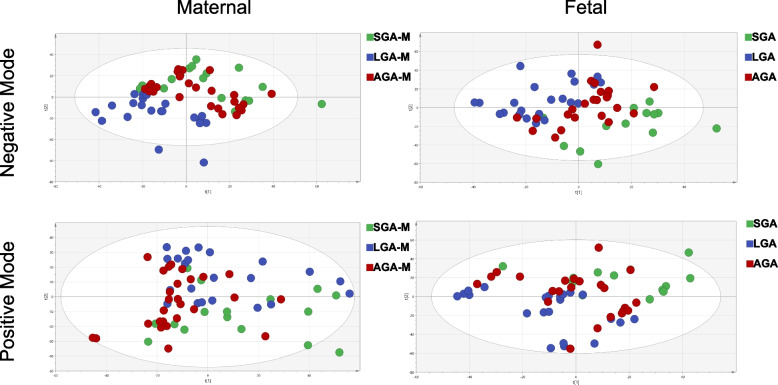
PLS-DA score plot for maternal and fetal metabolome under negative and positive MS mode

Supervised PLS-DA analysis was performed to investigate the metabolite profiles of each group, obtaining improved and better discrimination (Fig. 1). The metabolites that contributed to the separation of the SGA and AGA groups in the mother and their offspring were selected by the VIP value (> 1) calculated from the OPLS-DA model (Figure S1 and S2, Table S4), as well as those metabolites for the LGA and AGA comparison. R^2^ and Q^2^ values for SGA vs AGA maternal metabolome are 0.857 and -0.569 (Negative mass mode), 0.943 and -0.119(Positive mass mode). R2 and Q2 values for SGA vs AGA fetal metabolome are 0.958 and 0.0121 (Neg), 0.921 and -0.57(Pos). R2 and Q2 values for LGA vs AGA maternal metabolome are 0.955 and 0.155 (Neg), 0.959 and -0.104(Pos). R2 and Q2 values for LGA vs AGA fetal metabolome are 0.944 and 0.0407 (Neg), 0.887 and -0.361(Pos).

**Fig. 2:**
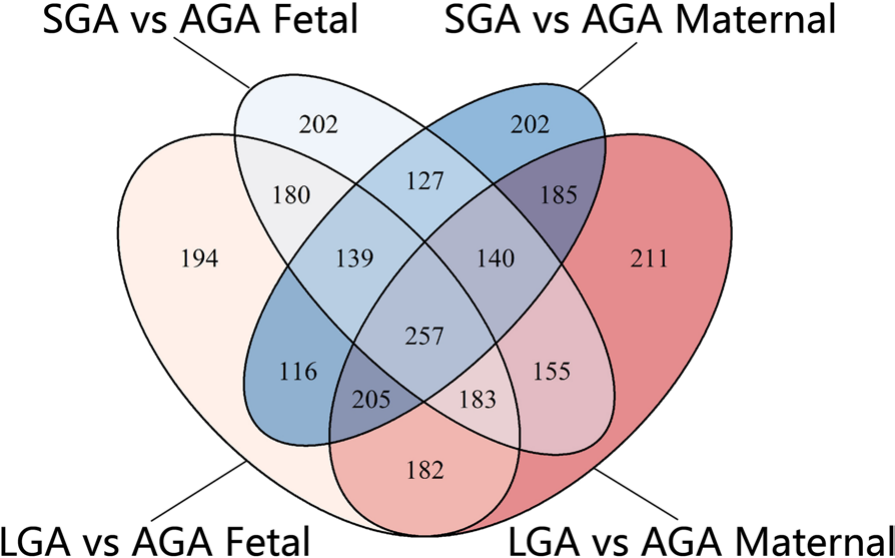
Venn diagram showing the identified metabolites selected by the OPLS-DA models

From the Venn diagram (Fig. 2), we identified 1518 and 1456 metabolites that contributed to the separation of LGA versus AGA mothers and their offspring (differential metabolites, DMs), respectively, and mothers and their offspring shared 827 DMs. Meanwhile, there were 1371 and 1383 metabolites that attribute to the separation of SGA versus AGA mother and offspring, respectively, and mothers and their offspring share 663 DMs. Overlapping of these 4 kinds of DMs yielded 257 metabolites that represent the same DMs shared by both the SGA and LGA groups when compared with the AGA group.

**Fig. 3:**
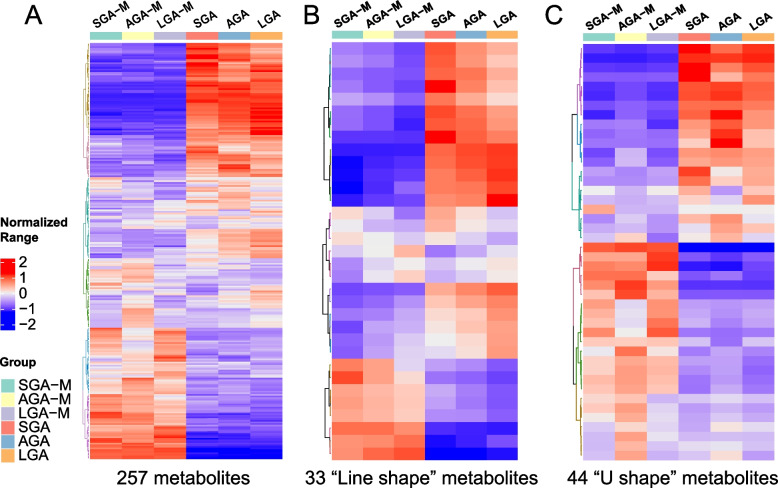
Heatmap showing normalized metabolite abundance in different groups. **A** The 257 metabolites shared by the SGA and LGA groups and different from the AGA group.; **B** The 33 line-shaped metabolites display the increasing/decreasing abundance in the SGA/AGA/LGA group according to the newborn birthweight. **C** The 44 U-shaped metabolites display higher or lower abundance in both the SGA and LGA groups

Among these 257 metabolites (MS details are shown in Supplementary Table S4), we identified 2 types of metabolites from the heatmap (Fig. 3A), including 33 “line-shaped” metabolites (Fig. 3B) and 44 “U-shaped” metabolites (Fig. 3C). The fold change between SGA and AGA, or LGA and AGA was calculated (Table S4), and the U-shaped metabolites were higher or lower in both SGA/LGA mothers and babies (means fold change of SGA/AGA and LGA/AGA are both > 1 or < 1), while line-shaped metabolites were increased or decreased among SGA/AGA/LGA mothers and babies (means fold change of SGA/AGA > 1 and LGA/AGA < 1, or fold change of SGA/AGA < 1 and LGA/AGA > 1)..

We put 33 line-shaped and 44 U-shaped metabolites into pathway analysis, respectively. The topological impact factors and enrichment analysis p-values of the corresponding pathways are shown in the bubble graph (Fig. S1). The results suggested that U-shaped metabolites were enriched in linoleic acid metabolism (metabolites including Phosphatidylcholine-PC), arachidonic acid metabolism (metabolites including PC, Prostaglandin E2), and glycerophospholipid metabolism (metabolites including PC, Citicoline) (Fig. S3A). Line-shaped metabolites were enriched in glycerophospholipid metabolism (metabolites including phosphatidylethanolamine-PE, PC, 1-Acyl-sn-glycero-3-phosphocholine-LysoPC), alpha-linolenic acid metabolism (metabolites including PC, tetracosanoic acid, 13(S)-HODE), and linoleic acid metabolism (metabolites including PC) (Fig. S3B).

### Metabolites associated with newborn birthweight

**Fig. 4:**
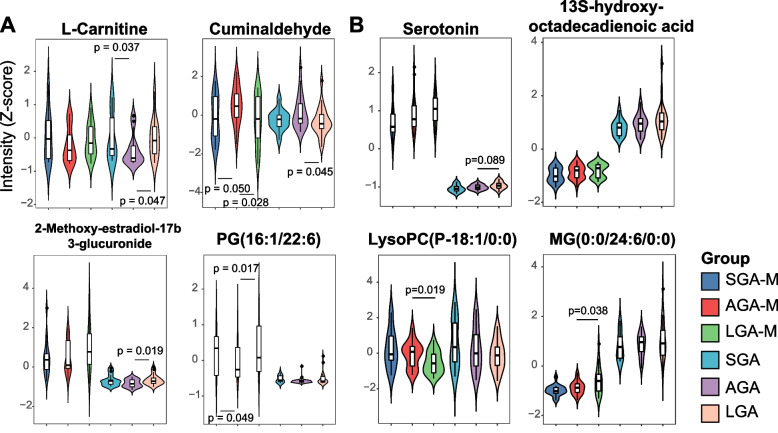
Abundance of metabolites in different groups. **A** Representative U-shapes metabolites; **B** Representative line-shaped metabolites; Abundance was normalized using Z score transformation of intensity

As shown in Fig. 4A, the U-shaped metabolites were more highly expressed in both SGA and LGA maternal and cord blood, including L-carnitine, PG(16:1/ 22:6) (denoted PG) and 2-methoxy-estradiol-17b 3-glucuronide (denoted glucuronide). However, cuminaldehyde was significantly downregulated in the SGA and LGA groups.

As shown in Fig. 4B, line-shaped metabolites, including serotonin, 13S-hydroxyoctadecadienoic acid (denoted as 13(S)-HODE) and MG(0:0/24:6/0:0) (denoted as MG), showed increasing trends in both mothers and babies among the SGA, AGA and LGA groups. LysoPC(p-18:1/0:0) (denoted as LysoPC) showed decreasing trends in both mothers and babies among the SGA, AGA and LGA groups.

### Correlation between maternal and fetal metabolite abundance

**Fig. 5:**
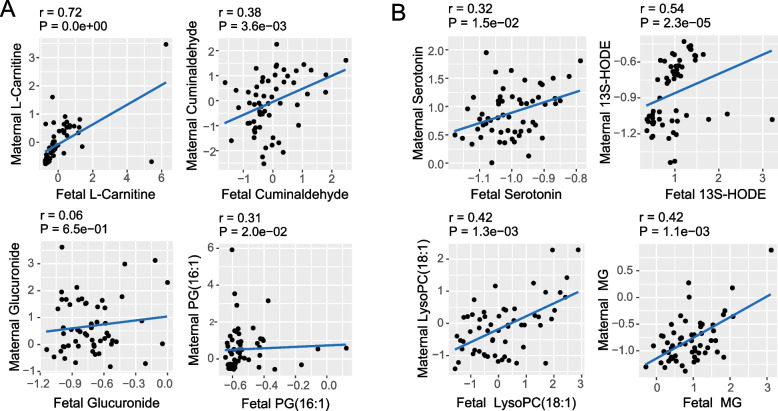
Association of metabolites in maternal and cord blood. **A** Representative U-shaped metabolites; **B** Representative line-shaped metabolites

The correlation between the maternal and fetal metabolome was analyzed using the Spearman method (Fig. 5). Among U-shaped metabolites, L-carnitine (*r* = 0.72, *p* < 0.001), cuminaldehyde (*r* = 0.38, *p* = 0.004) and PG (*r* = 0.31, *p* = 0.02) in maternal and cord blood were significantly positively correlated. Among line-shaped metabolites, serotonin (*r* = 0.32, *p* < 0.001), 13(S)-HODE (*r* = 0.54, *p* < 0.001), LysoPC (*r* = 0.42, *p* = 0.001), and monoacylglyceride (*r* = 0.42, *p* = 0.001) in maternal and cord blood were significantly positively correlated.

### Correlation between metabolite levels and clinical characteristics

To further investigate the association between the metabolome and clinical characteristics, partial correlation analysis was performed (Table 2 and Table 3). Among the U-shaped metabolites, cuminaldehyde was expressed at low levels in the SGA and LGA groups, and its abundance in maternal blood was positively correlated with maternal adiponectin (*r* = 0.320 *p* = 0.010) and HDL-C (adjusted *r* = 0.323 *p* = 0.011). Cuminaldehyde in cord blood was negatively correlated with maternal prepregnancy BMI (adjusted *r* = -0.356 *p* = 0.009) and weight gain (*r* = -0.267 *p* = 0.043), as shown in Table S2. L-Carnitine, PG and glucuronide were higher in the SGA and LGA groups. Maternal glucuronide was positively correlated with maternal HCY (adjusted *r* = 0.396, *p* = 0.002) and FFA (adjusted *r* = 0.479, *p*** < **0.001). Maternal glucuronide was negatively correlated with FBG (adjusted *r* = -0.360, *p* = 0.005) and HDL-C (adjusted *r* = -0.247, *p* = 0.058). Maternal PG was positively correlated with prepregnancy BMI (adjusted *r* = 0.328 *p* = 0.011). Cord blood PG was positively correlated with FFA (adjusted *r* = 0.285 *p* = 0.045).

**Table 2:** Correlation of U-shaped metabolites and blood index in maternal blood

		**L-Carnitine**		**Cuminaldehyde**		**2-methoxy-estradiol-17b 3-glucuronide**			
		**r**	***P***	**r**	***P***	**r**	***P***	**r**	***P***
**BMI**	Model1	-0.005	0.971	-0.179	0.154	0.177	0.158	**0.280**	**0.024**
	Model2	0.017	0.899	-0.180	0.169	0.110	0.403	**0.328**	**0.011**
**Weight Gain**	Model1	-0.140	0.267	**-0.252**	**0.043**	0.146	0.245	0.224	0.073
	Model2	-0.200	0.125	-0.240	0.065	0.071	0.591	-0.099	0.452
**HbA1c**	Model1	-0.099	0.496	-0.226	0.115	0.101	0.486	-0.164	0.255
	Model2	-0.149	0.329	-0.153	0.316	-0.091	0.554	-0.075	0.625
**FBG**	Model1	0.200	0.113	0.030	0.815	**-0.424**	** < 0.001**	-0.009	0.943
	Model2	0.205	0.119	0.036	0.788	**-0.360**	**0.005**	0.126	0.341
**HDL-C**	Model1	-0.064	0.610	0.242	0.052	**-0.254**	**0.041**	-0.110	0.384
	Model2	-0.026	0.845	**0.329**	**0.010**	-0.247	0.058	-0.124	0.347
**LDL-C**	Model1	0.052	0.681	0.067	0.599	-0.070	0.581	-0.204	0.103
	Model2	0.021	0.872	0.081	0.536	-0.116	0.378	**-0.276**	**0.033**
**FFA**	Model1	0.046	0.714	-0.150	0.234	**0.424**	** < 0.001**	-0.196	0.118
	Model2	0.080	0.544	-0.140	0.287	**0.479**	** < 0.001**	0.044	0.740
**Leptin**	Model1	0.098	0.440	-0.131	0.303	-0.165	0.192	**0.285**	**0.022**
	Model2	-0.023	0.861	-0.222	0.091	-0.254	0.052	0.051	0.702
**Adiponectin**	Model1	0.033	0.795	**0.320**	**0.010**	-0.185	0.143	-0.052	0.682
	Model2	0.076	0.567	0.064	0.628	0.006	0.962	0.052	0.696
**HCY**	Model1	-0.242	0.052	**-0.316**	**0.010**	**0.440**	** < 0.001**	-0.150	0.234
	Model2	-0.226	0.082	-0.102	0.438	**0.396**	**0.002**	-0.084	0.523

Model 1 is non-adjusted

Model 2 is adjusted for maternal age, parity, GDM and gestational age, and fetal sex

**Table 3:** Correlation of line-shaped metabolites and blood index in maternal blood

		**Serotonin**		**13(S)-HODE**		**LysoPC(P-18:1)**		**MG(0:0/24:6)**	
		**r**	***P***	**r**	***P***	**r**	***P***	**r**	***P***
**BMI**	Model1	**0.259**	**0.037**	**0.462**	** < 0.001**	-0.077	0.544	0.024	0.848
	Model2	0.193	0.140	0.441	** < 0.001**	-0.073	0.580	0.115	0.380
**Weight Gain**	Model1	0.191	0.128	**0.404**	**0.001**	-0.135	0.283	-0.120	0.341
	Model2	0.230	0.076	**0.400**	**0.002**	-0.133	0.312	-0.155	0.238
**HbA1c**	Model1	0.212	0.139	0.125	0.388	0.047	0.748	-0.055	0.705
	Model2	0.107	0.483	0.259	0.086	0.060	0.693	-0.084	0.583
**FBG**	Model1	-0.227	0.071	-0.157	0.216	**0.248**	**0.048**	0.073	0.567
	Model2	-0.188	0.155	-0.129	0.328	0.186	0.159	0.033	0.805
**HDL-C**	Model1	-0.190	0.129	-0.139	0.268	**0.286**	**0.021**	-0.073	0.563
	Model2	-0.236	0.069	-0.152	0.247	0.192	0.142	-0.145	0.270
**LDL-C**	Model1	-0.076	0.545	-0.152	0.228	0.166	0.185	0.118	0.350
	Model2	0.016	0.906	-0.041	0.758	0.216	0.098	0.044	0.741
**FFA**	Model1	**0.312**	**0.011**	0.069	0.583	-0.111	0.380	**0.313**	**0.011**
	Model2	0.303	0.019	0.052	0.695	-0.150	0.251	**0.315**	**0.014**
**Leptin**	Model1	-0.010	0.937	0.170	0.181	-0.084	0.511	-0.182	0.151
	Model2	-0.045	0.735	0.092	0.487	-0.013	0.920	-0.180	0.172
**Adiponectin**	Model1	-0.126	0.321	**-0.284**	**0.023**	0.097	0.445	-0.048	0.706
	Model2	0.000	0.997	-0.079	0.552	0.174	0.187	-0.018	0.893
**HCY**	Model1	**0.361**	**0.003**	**0.258**	**0.038**	-0.118	0.350	-0.032	0.800
	Model2	0.244	0.061	0.111	0.397	-0.211	0.106	-0.075	0.571

Model 1 is non-adjusted

Model 2 is adjusted for maternal age, parity, GDM and gestational age, and fetal sex

Among the line-shaped metabolites, serotonin, 13(S)-HODE and MG showed increasing trends in the SGA, AGA and LGA groups. The abundance of serotonin in maternal blood was significantly positively correlated with prepregnancy BMI (*r* = 0.259, *p* = 0.037) and maternal HCY (*r* = 0.361, *p* = 0.003). Cord blood serotonin levels were also significantly positively correlated with prepregnancy BMI (adjusted *r* = 0.354, *p* = 0.009) and cord blood glucose (*r* = -0.355, *p* = 0.008). Maternal 13(S)-HODE was positively correlated with maternal prepregnancy BMI (adjusted *r* = 0.441, *p* < 0.001), weight gain (adjusted *r* = 0.400, *p* = 0.002), and maternal HCY (*r* = 0.258, *p* = 0.038) and negatively correlated with maternal adiponectin (*r* = -0.28, *p* = 0.023). Moreover, maternal MG was positively related to maternal FFA (adjusted *r* = 0.315, *p* = 0.014). Maternal LysoPC showed decreasing trends in the SGA, AGA and LGA groups that were positively related to maternal HDL-C (*r* = 0.286, *p* = 0.021), as well as LysoPC in cord blood with cord HDL-C (*r* = 0.263, *p* = 0.050).

## Discussion

Previous studies in the HAPO and other cohorts have demonstrated the effect of maternal obesity and gestational diabetes mellitus (GDM) on newborn size using metabolomic technology, suggesting that the intrauterine environment provided by mothers could affect newborn outcomes [24–26]. Our study group discovered that lower birth weight is an independent risk factor for later diabetes or IGT and showed for the first time that this risk factor also applies to a Chinese population [4], and both intrauterine under- and overnutritional status could affect insulin resistance in adulthood in animal experiments [27, 28], which was consistent with the developmental origins of health and disease (DoHAD) hypothesis. In this study, we aim to provide a new perspective from the view of newborns to discover the association of the metabolome between SGA, AGA, and LGA newborn babies and their mothers.

In this study, using an untargeted metabolomics approach, we show for the first time that specific metabolites were associated with both SGA and LGA mothers and their offspring, which might explain the phenomenon that children born with SGA and LGA had similar adverse outcomes. We observed that U-shaped metabolites were enriched in linoleic acid, arachidonic acid and glycerophospholipid metabolism. The metabolites associated with the top 3 pathways were PC, citicoline and Prostaglandin E2. The importance of PC regulating lipid, lipoprotein and whole-body energy metabolism has been demonstrated in numerous dietary studies and knockout animal models [29]. For example, PC is an essential component of the very low-density lipoprotein (VLDL) complex [29], and the inhibition of hepatic PC synthesis impairs VLDL secretion and is linked to fatty liver disease in rodents as well as in human [30–32]. Related compounds of PC metabolic pathway, PC(14:0/22:5), PC(22:4/P-18:0), PG(16:1), and citicoline showed decreasing or increasing trends in both SGA/LGA maternal and fetal blood, which may indicate a disturbance of PC metabolism in these mother–offspring pairs.

In addition to pathway analysis, we screened 44 metabolites by p value, and identified that cuminaldehyde is lower in the SGA and LGA groups and is reported to be a volatile chemical constituent of cumin seed [33]. It has been reported that cuminaldehyde presents in trace amounts in the blood and milk of ewes fed with cumin seed [34], and its molecular weight is 148.205 Da, indicating that cuminaldehyde should be easily transported across the placental barrier. Cuminaldehyde was reported to have an inhibitory effect in vitro against rat lens aldose reductase and alpha-glucosidase (human metabolome database) and to protect against nonalcoholic fatty liver disease in rats fed a high-fat diet [35]. This inhibitory action of cuminaldehyde suggests its potential utility as an antidiabetic therapeutic [36], and the potential mechanism might be associated with its insulinotropic and ß-cell protective action [37]. In our study, cuminaldehyde was also significantly positively correlated with maternal adiponectin levels, negatively correlated with maternal BMI before pregnancy, and weight gain during pregnancy. These data indicate that cuminaldehyde is involved in lipid metabolism. According to previous reports, cuminaldehyde shows its superior activity for lipoxygenase (LOX) inhibitor, thereby blocking the oxidation of unsaturated fatty acids and inhibiting reactive oxygen species (ROS) production [33]. It is also quite interesting that adiponectin also showed a reverse effect against LOX-1 for reducing ROS production [38]. Therefore, due its anti-diabetic, anti-hepatotoxic and lipid metabolism modulating abilities, the low abundance of cuminaldehyde in both SGA/LGA maternal and neonatal blood might contribute to their increased metabolic risk.

2-Methoxy-estradiol-17b 3-glucuronide was generated when UDP-glucuronate was transferred to UDP and was enriched in the SGA and LGA groups. Glucose is phosphorylated to glucose 6-phosphate in hepatocytes, which then produces UDP-glucose. UDP-glucose can be used to synthesize glycogen, UDP-glucuronate and UDP-galactose [39]. Even though the glucose in maternal and fetal blood showed no difference in the SGA, AGA and LGA groups in our study, the increased level of 2-methoxy-estradiol-17b 3-glucuronide in the SGA/LGA groups might indicate a higher hepatic UDP-glucuronate transition rate in SGA and LGA newborns and their mothers. Recent research has also found that glucuronidation of bilirubin, drugs and xenobiotics in hepatocytes by glucuronide is considered a detoxification process [40]. Thus, the alteration in glucuronides concentrations might lead to impairment of the detoxification biological processes. Consistent with our hypothesis, abnormal glucuronide concentrations predict gestational diabetes in early pregnancy [41]. We also observed that the glucuronide was positively related with FFA in maternal blood, and negatively related with adiponectin in cord blood. Fatty acids have been reported to inhibit glucuronidation of different substrates [42, 43], which might explain the link of glucuronide to lipid index. These initial results are suggestive of a link between the abnormal abundance of certain metabolites in the intrauterine environment and the higher metabolic disorder development rate of both SGA and LGA newborns.

From the pathway analysis of line-shaped metabolites, we found out that top 3 pathways are all related to a series of phospholipids and fatty acids metabolites, including PC, PE, LysoPC and fatty acids. Since line-shaped metabolites are positively or negatively correlated to newborn birthweight, we suppose that it might closely related to lipogenesis. The PC and PE are well studied for the role in lipid droplet formation [44, 45] and de novo lipogenesis regulation [46]. Therefore, it is plausible that the line-shaped metabolites are enriched in phospholipid metabolism pathways.

We also discover that serotonin, which was produced within the central nervous system and is in charge of regulating behavior, suppressing appetite and promoting energy expenditure, showed increasing trends [47]. Moreover, serotonin and insulin are colocalized in secretory beta-granules and are cosecreted by the stimulation of glucose [48]. Serotonin in adipose tissue promotes adipogenesis in white adipocytes, and elevated serotonin levels are associated with obesity. In our study, serotonin in maternal and fetal blood was also positively related to maternal BMI before pregnancy and the conventional biochemical index FFA, which indicated that the birthweight of newborns was possibly associated with maternal energy homeostasis.

13S-hydroxyoctadecadienoic acid, known as 13(S)-HODE, is the major lipoxygenation product synthesized in the body from linoleic acid and has been proposed as a biomarker for evaluating oxidative stress [49]. 13(S)-HODE has been reported to be increased in T2DM and alcohol-induced liver injury mouse models [50]. In our study, the level of 13(S)-HODE in maternal and cord blood showed increasing trends in the SGA, AGA and LGA groups and was significantly positively correlated with maternal prepregnancy BMI, weight gain, and HCY. Hyperhomocysteinemia is an independent risk factor for cardiovascular diseases and can also activate oxidative stress in endothelial cells [51]. Our results suggest that this oxidative stress marker, 13(S)-HODE, is positively correlated with neonatal birthweight, and related to maternal weight issues.

An additional interest in our study was the association of the maternal and fetal metabolome, which might explain the molecular mechanism underlying the DoHAD hypothesis of how the intrauterine environment regulates fetal metabolism. From the HAPO study [24], we can also tell that amino acids and acylcarnitine demonstrated significant correlations between maternal and cord blood levels via a series of transporters. Our analyses demonstrated that most of the metabolites in maternal blood showed a significant correlation with those in cord blood, including small molecule metabolites, such as the small polar molecules cuminaldehyde and serotonin, lipophilic molecule PG and 13(S)-HODE. Since most exchange across the placenta is driven by diffusion or specific transport proteins, small polar molecules and lipophilic substances dissolve readily through the entire syncytiotrophoblast plasma membrane and easily enter the fetal circulation system [18]. The same alterations of maternal and fetal metabolome were also found in the preeclampsia pregnant groups, probably due to the metabolites passive transfer of metabolites across the placenta [52]. It has been reported that compounds, such as reactive oxygen species, which could reflect maternal metabolic status, may cross the placenta and affect the growing fetus in a similar fashion [53]. Therefore, some of the metabolites in cord blood are derived from transplacental transfer and are significantly influenced by maternal blood metabolite levels. Moreover, diffused metabolites could have a role in regulating fetal metabolism, which warrants further investigation. Despite differences in metabolomics, recent studies show that umbilical artery parameter is also correlated with newborn birthweight [54], especially in gestational diabetic mothers [55]. Therefore, we suppose that both maternal blood metabolites and umbilical artery hemodynamic factors influence birth weight.

This study had several strengths. An untargeted metabolomic assay was conducted to profile the whole picture of metabolites in both peripheral blood of pregnant women and cord blood of their babies, which could more extensively focus on molecular transfer in the intrauterine environment. We show for the first time that specific metabolites were associated with both SGA and LGA mothers and their offspring, which might explain why the children born with SGA and LGA had similar adverse outcomes. To further explore the clinical relevance of these metabolites, the correlation of maternal and fetal metabolomes with their phenotype was analyzed, and we found that maternal BMI, weight gain and adipokines, instead of blood glucose indices, were significantly associated with metabolite levels. The association of the maternal and fetal metabolome was also analyzed, and fetal metabolites in cord blood were significantly related to maternal blood metabolite levels. One limitation is that the confidence of metabolites annotation via nontargeted assays is limited, therefore the accurate quantification of our discovered metabolites need to be further confirmed by targeted assays and using standards for exact identification and quantitation. Another limitation is that the sample size of this study is relatively small. A larger cohort study with dietary records and animal models are planned to test these hypotheses.

In conclusion, we demonstrated a broad-scale association of metabolites between pregnant women and their offspring. We found 2 types of metabolites that changed with different patterns according to newborn birthweight. Among these metabolites, cuminaldehyde and glucuronide, denoted as “U-shaped” metabolites, associated with both the SGA and LGA groups have been reported to participate in glucose regulation, which might explain the phenomenon where these children had similar adverse outcomes in adulthood. Serotonin and 13(S)-HODE are denoted as “line-shaped” metabolites, which correlate positively with newborn birthweight and are involved in energy homeostasis regulation and oxidative stress. These investigations demonstrate broad-scale metabolic differences related to newborn birthweight that help to provide new insights into insulin resistance and risks of metabolic syndrome in adults with SGA and LGA babies and might identify potential new markers for adverse newborn outcomes in pregnant women.

## Supplementary Information

**Additional file 1: Figure S1.** OPLS-DA for negative mode.

**Additional file 2: Figure S2.** OPLS-DA for positive mode.

**Additional file 3: Figure S3.** Pathway analysis of (A) U-shaped metabolites and (B) line-shaped metabolites.

**Additional file 4: Table S1.** Characteristics of mothers and their offspring. **Table S2.** Correlation of U-shape metabolites and blood index in cord blood. **Table S3.** Correlation of Line-shape metabolites and blood index in cord blood.

**Additional file 5: Table S4.** Metabolites and pathway information.

## Data Availability

The raw metabolomics data of the current study would be available from the corresponding author on reasonable request.

## Abbreviations

SGA: Small-for-gestational
AGA: Appropriate-for-gestational
LGA: Large-for-gestational
BMI: Body mass index
MS: Mass spectrometers
PLS-DA: Partial least squares regression discriminant analysis
OPLS-DA: Orthogonal partial least squares regression discriminant analysis
VIP: Variable importance in projection
DMs: Differential metabolites
