# Supplementary figures and images for: Nontargeted metabolomics reveals the potential mechanism underlying the association between birthweight and metabolic disturbances
Source: BMC Pregnancy Childbirth. 2023 Jan 9;23:14. doi: 10.1186/s12884-023-05346-6 (PMC9830726; doi:10.1186/s12884-023-05346-6)

Maternal

LGA VS AGA

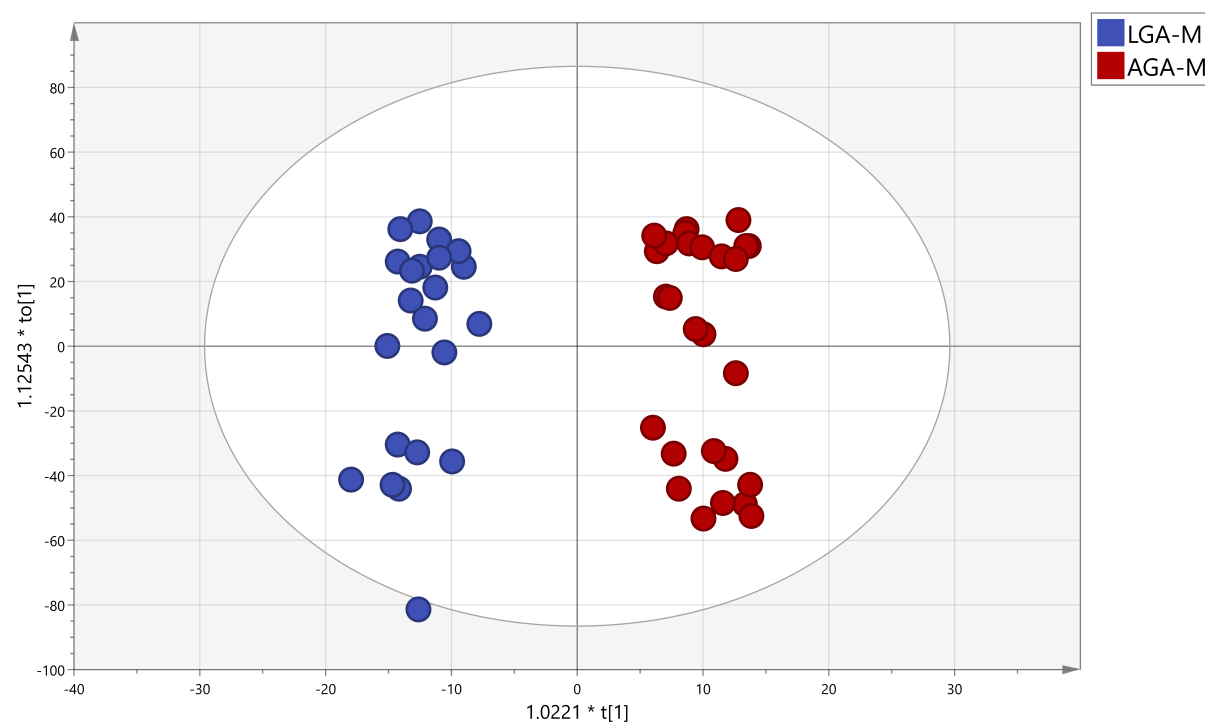

SGA VS AGA

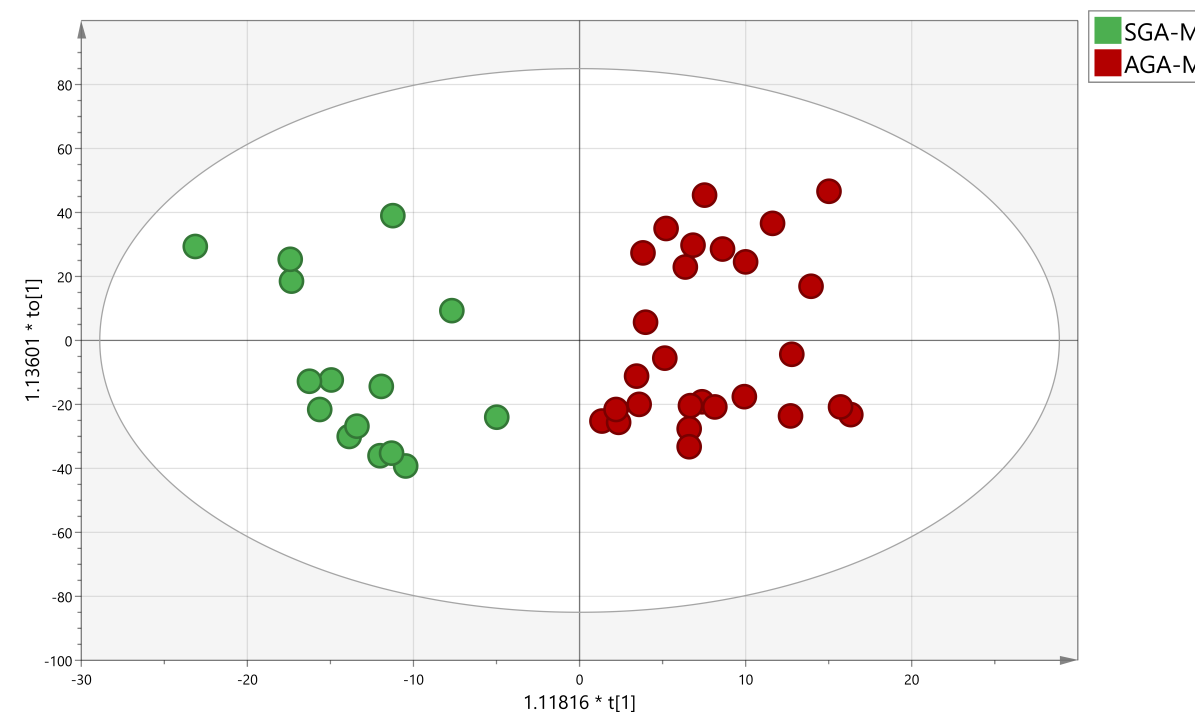

Fetal

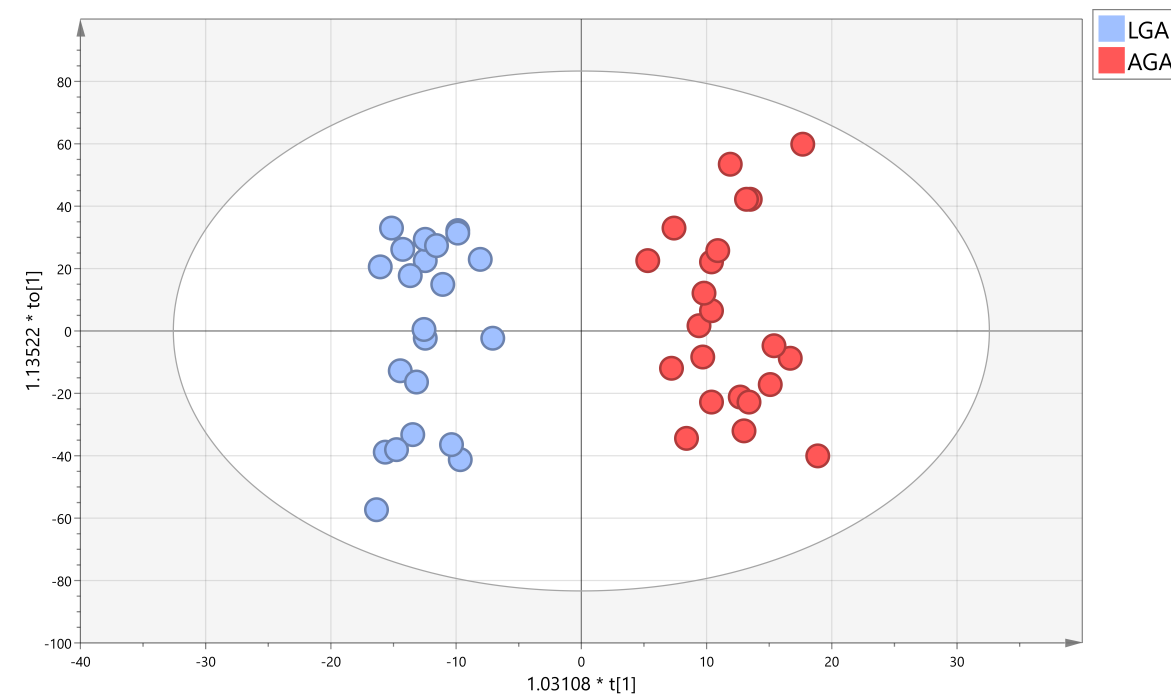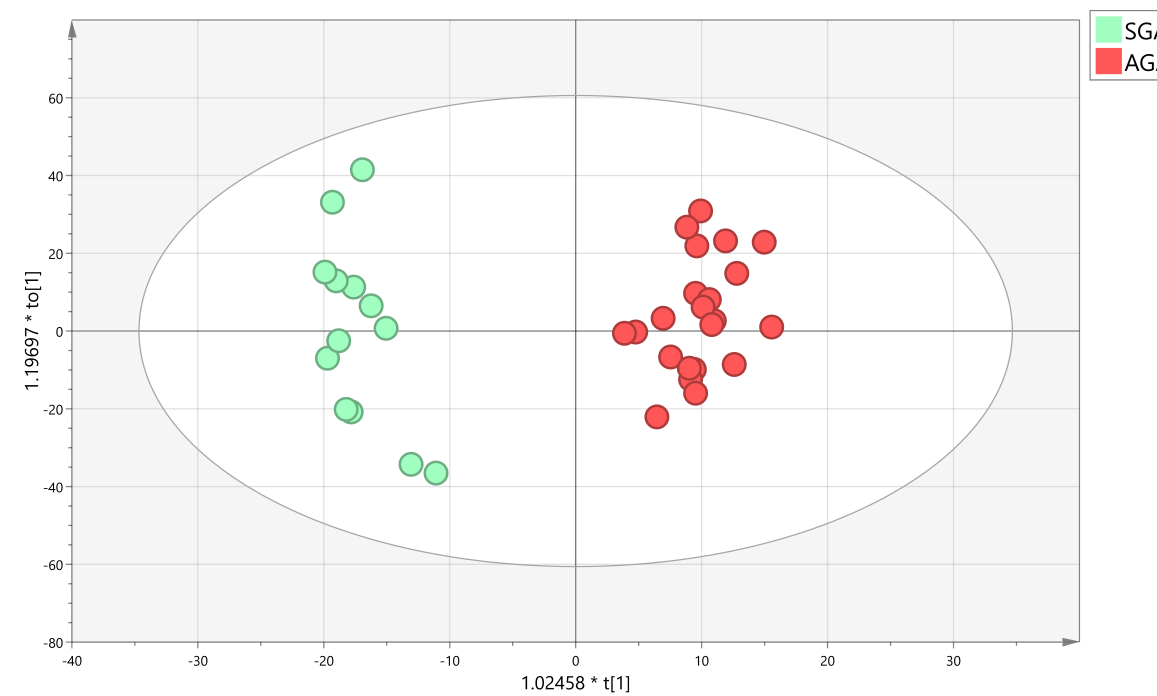

Figure S1. OPLS-DA for negative mode

Supplement: Supplementary file 1 — Additional file 1: Figure S1. OPLS-DA for negative mode. [file 12884_2023_5346_MOESM1_ESM.pdf]

**Maternal**

**LGA VS AGA**

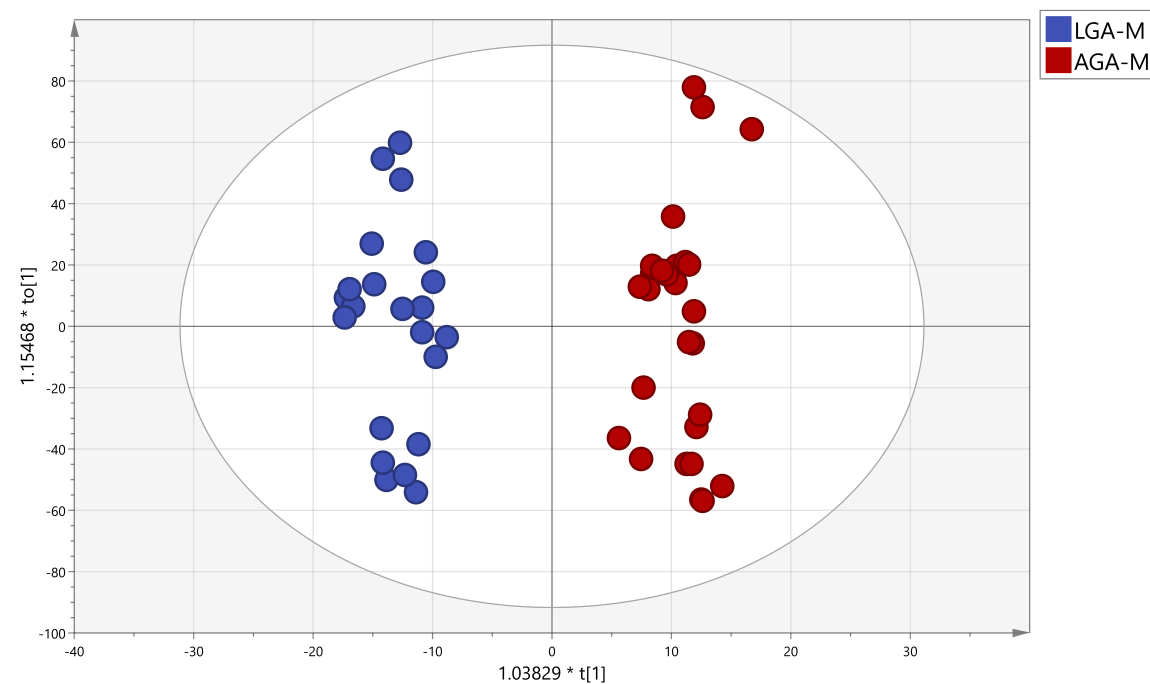

**SGA VS AGA**

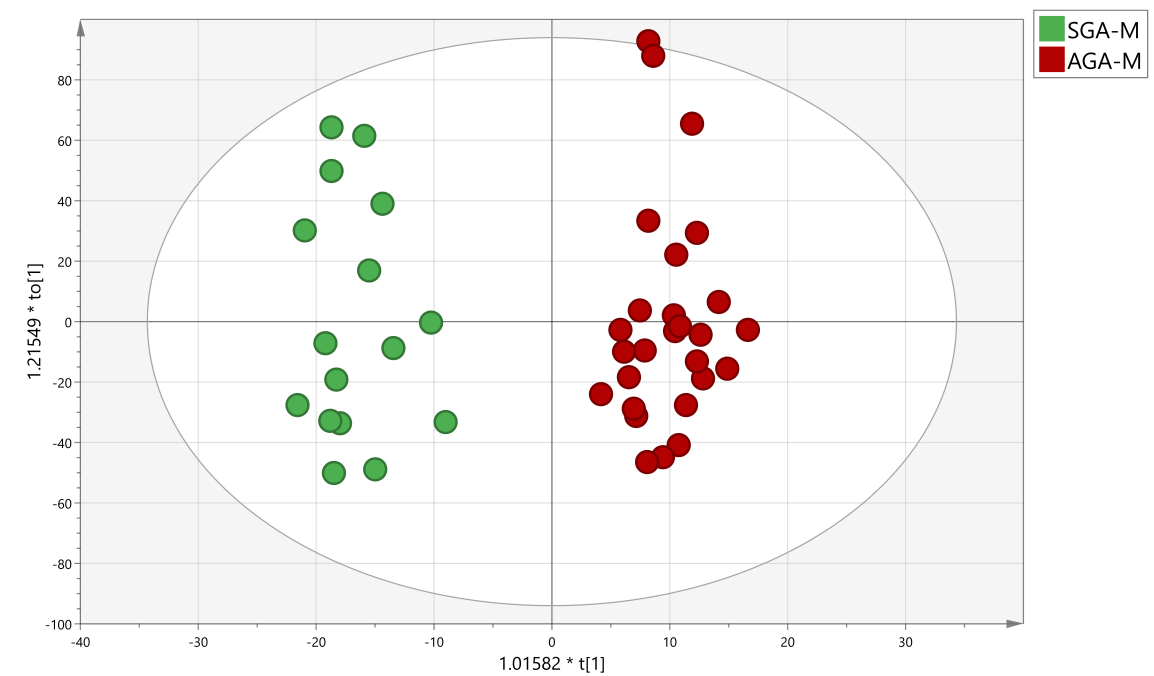

**Fetal**

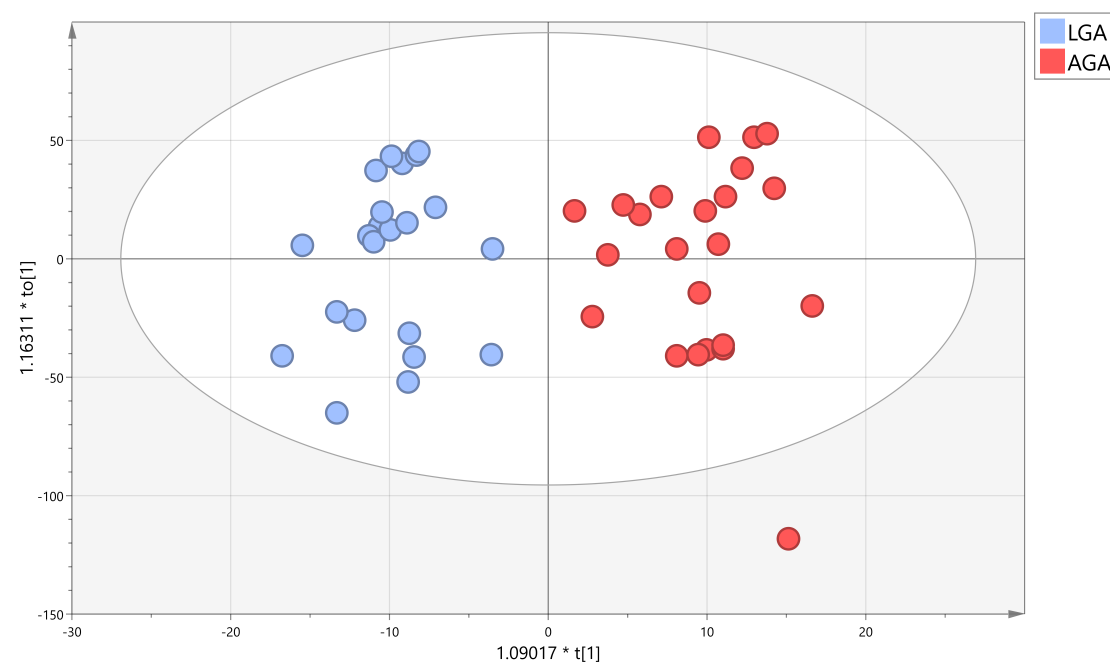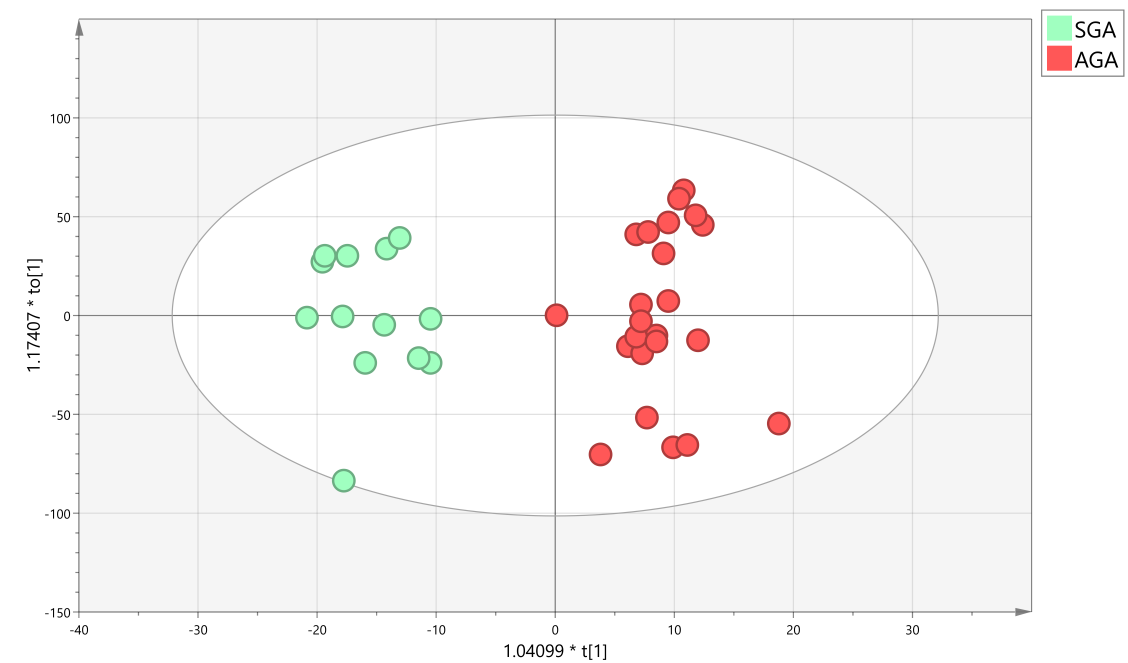

**Figure S2. OPLS-DA for positive mode**

Supplement: Supplementary file 2 — Additional file 2: Figure S2. OPLS-DA for positive mode. [file 12884_2023_5346_MOESM2_ESM.pdf]

**A**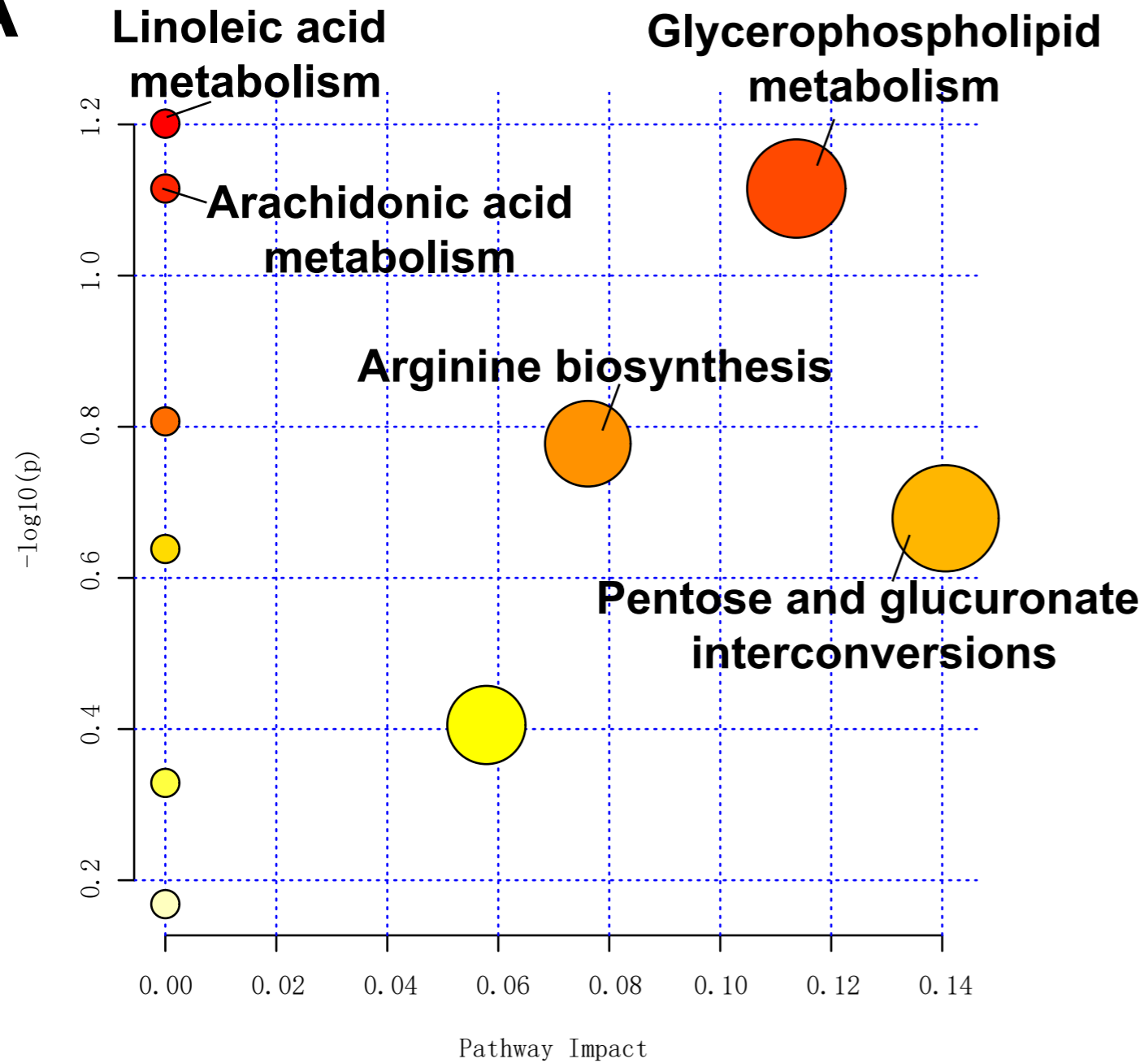**B**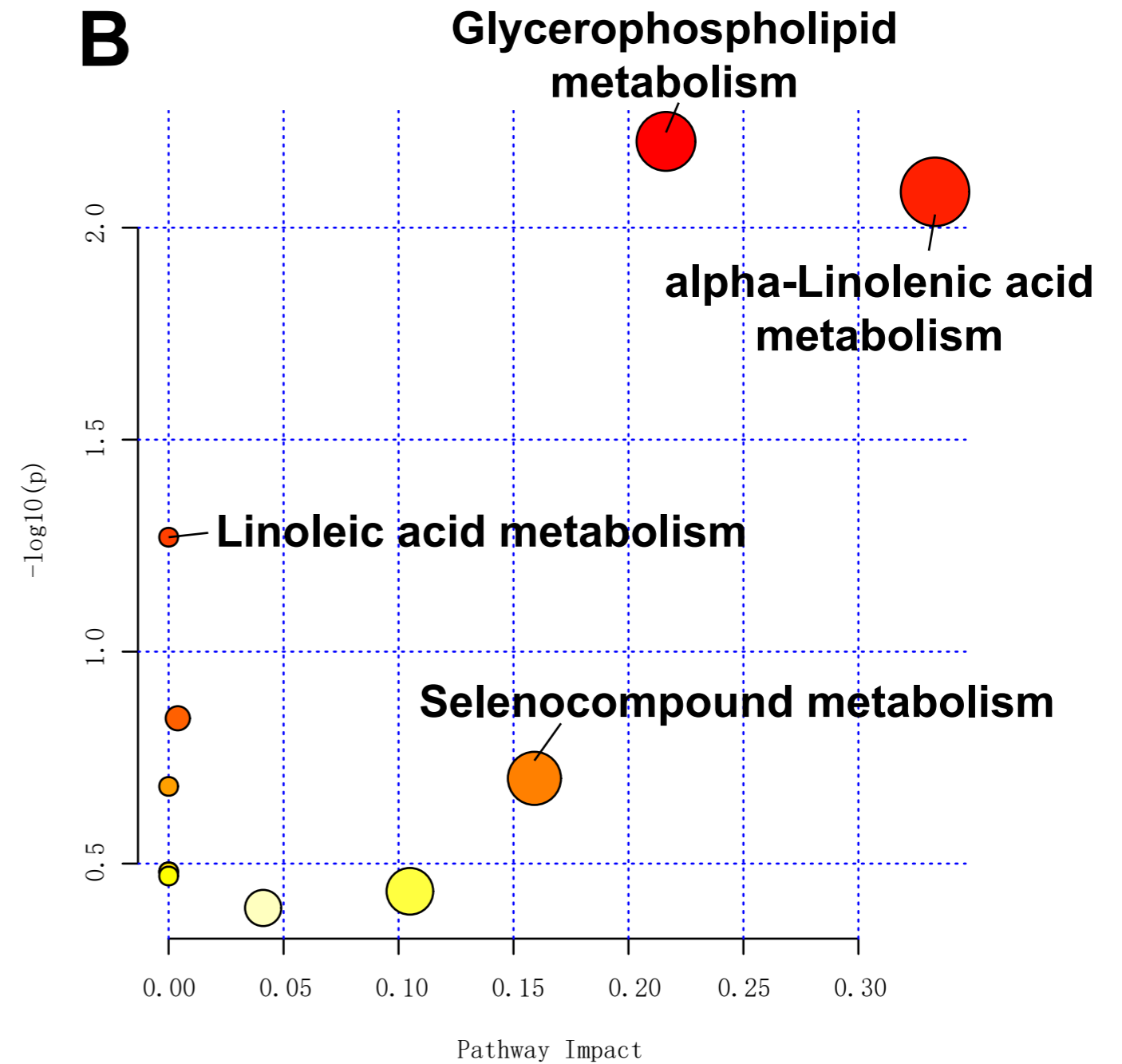

Figure S3. Pathway analysis of (A) U-shaped metabolites and (B) line-shaped metabolites

Supplement: Supplementary file 3 — Additional file 3: Figure S3. Pathway analysis of (A) U-shaped metabolites and (B) line-shaped metabolites. [file 12884_2023_5346_MOESM3_ESM.pdf]
