# Supplementary material for: Nontargeted metabolomics reveals the potential mechanism underlying the association between birthweight and metabolic disturbances
Source: BMC Pregnancy Childbirth. 2023 Jan 9;23:14. doi: 10.1186/s12884-023-05346-6 (PMC9830726; doi:10.1186/s12884-023-05346-6)
Supplement: Supplementary file 4 — Additional file 4: Table S1. Characteristics of mothers and their offspring. Table S2. Correlation of U-shape metabolites and blood index in cord blood. Table S3. Correlation of Line-shape metabolites and blood index in cord blood. [file 12884_2023_5346_MOESM4_ESM.docx]

**Supplementary Tables**

TableS1 Characteristics of mothers and their offspring

| Characteristic | SGA | AGA | LGA | *P* |
| --- | --- | --- | --- | --- |
| *n* | 16 | 28 | 23 |  |
| Maternal Characteristics, Mean(SD) |  |  |  |  |
| HbA1c(%) | 5.4 (0.3) | 5.4 (0.5) | 5.5 (0.7) | 0.83 |
| HCY (μmol/L) | 38.7 (1.1) | 38.4 (0.9) | 39.0 (1.1) | 0.96 |
| GA (%) | 11.4 (3.2) | 12.9 (1.5) | 11.4 (2.9) | 0.08 |
| TC (mmol/L) | 6.0 (1.1) | 6.0 (1.5) | 5.5 (1.4) | 0.44 |
| TG (mmol/L) | 2.8 (0.9) | 3.2 (1.5) | 4.2 (2.6) | 0.06 |
| HDL-C (mmol/L) | 1.7 (0.4) | 1.8 (0.4) | 1.4 (0.3) | **0.01** |
| LDL-C (mmol/L) | 3.2 (0.8) | 3.2 (0.7) | 2.7 (1.0) | **0.04** |
| Lp(a) (mg/L) | 158.9 (229.4) | 108.6 (93.6) | 96.4 (116.3) | 0.40 |
| FFA (μmol/L) | 562.6 (200.4) | 567.9 (221.8) | 669.7 (314.8) | 0.30 |
| hs-CRP (mg/L) | 3.4 (1.6) | 3.7 (3.6) | 5.2 (3.9) | 0.19 |
| Newborn Characteristics, Mean(SD) |  |  |  |  |
| Cord TC (mmol/L) | 1.7 (0.6) | 1.8 (0.6) | 2.0 (1.1) | 0.59 |
| Cord TG (mmol/L) | 0.2 (0.1) | 0.2 (0.3) | 0.4 (1.0) | 0.47 |
| Cord HDL-C (mmol/L) | 0.7 (0.2) | 0.7 (0.2) | 0.8 (0.3) | 0.40 |
| Cord LDL-C (mmol/L) | 0.7 (0.3) | 0.7 (0.5) | 0.7 (0.7) | 0.91 |
| Cord Lp(a) (mg/L) | 14.7 (20.3) | 9.6 (8.7) | 20.0 (40.9) | 0.46 |
| Cord FFA (μmol/L) | 0.6 (0.2) | 1.1 (1.3) | 1.4 (0.9) | 0.89 |
| Cord hs-CRP (mg/L) | 0.2 (0.5) | 0.2 (0.6) | 2.8 (9.2) | 0.25 |

Data are n(%) or means±SD unless otherwise indicated. Categorical variable frequencies and continuous variable means were compared for SGA/AGA/LGA mothers using Fisher exact tests and One-way ANOVA, respectively

Table S2. Correlation of U-shape metabolites and blood index in cord blood

|  |  | L-Carnitine | | Cuminaldehyde | | 2-methoxy-estradiol-17b 3-glucuronide | | PG(16:1/22:6) | |
| --- | --- | --- | --- | --- | --- | --- | --- | --- | --- |
|  |  | **r** | ***P*** | **r** | ***P*** | **r** | ***P*** | **r** | ***P*** |
| Maternal BMI | Model1 | 0.081 | 0.545 | **-0.342** | **0.009** | 0.137 | 0.307 | **-0.260** | **0.049** |
|  | Model2 | 0.116 | 0.407 | **-0.356** | **0.009** | **0.305** | **0.027** | -0.120 | 0.391 |
| Maternal  Weight Gain | Model1 | 0.008 | 0.953 | **-0.267** | **0.043** | 0.160 | 0.231 | -0.140 | 0.293 |
|  | Model2 | -0.050 | 0.724 | -0.235 | 0.090 | 0.100 | 0.476 | -0.182 | 0.192 |
| Cord Glucose | Model1 | -0.102 | 0.464 | 0.117 | 0.398 | 0.084 | 0.544 | 0.26 | 0.058 |
|  | Model2 | -0.199 | 0.171 | 0.245 | 0.090 | -0.085 | 0.560 | -0.041 | 0.778 |
| Cord C-peptide | Model1 | -0.006 | 0.967 | -0.136 | 0.326 | 0.162 | 0.241 | -0.002 | 0.990 |
|  | Model2 | 0.186 | 0.200 | -0.075 | 0.607 | 0.274 | 0.056 | 0.055 | 0.710 |
| Cord HDL-C | Model1 | -0.127 | 0.357 | 0.181 | 0.186 | -0.076 | 0.580 | 0.224 | 0.099 |
|  | Model2 | -0.156 | 0.279 | 0.204 | 0.155 | 0.018 | 0.899 | **0.315** | **0.026** |
| Cord LDL-C | Model1 | 0.113 | 0.411 | 0.221 | 0.105 | -0.245 | 0.071 | 0.184 | 0.179 |
|  | Model2 | -0.058 | 0.688 | **0.282** | **0.047** | 0.102 | 0.480 | 0.272 | 0.056 |
| Cord FFA | Model1 | 0.059 | 0.667 | 0.130 | 0.344 | -0.067 | 0.627 | 0.181 | 0.186 |
|  | Model2 | -0.023 | 0.873 | 0.209 | 0.144 | 0.131 | 0.365 | **0.285** | **0.045** |
| Cord Leptin | Model1 | -0.017 | 0.901 | 0.060 | 0.656 | 0.107 | 0.428 | 0.096 | 0.479 |
|  | Model2 | 0.011 | 0.941 | 0.004 | 0.979 | -0.090 | 0.524 | 0.022 | 0.879 |
| Cord Adiponectin | Model1 | **-0.292** | **0.044** | -0.019 | 0.896 | -0.180 | 0.221 | -0.073 | 0.622 |
|  | Model2 | -0.068 | 0.666 | -0.116 | 0.461 | **-0.314** | **0.040** | 0.137 | 0.382 |

Model 1 is non-adjusted.

Model 2 is adjusted for maternal age, parity, GDM and gestational age, and fetal sex.

Table S3. Correlation of Line-shape metabolites and blood index in cord blood

|  |  | Serotonin | | 13S-HODE | | LysoPC(P-18:1) | | MG(0:0/24:6) | |
| --- | --- | --- | --- | --- | --- | --- | --- | --- | --- |
|  |  | **r** | ***P*** | **r** | ***P*** | **r** | ***P*** | **r** | ***P*** |
| Maternal BMI | Model1 | **0.347** | **0.008** | **0.462** | **<0.001** | -0.077 | 0.544 | 0.024 | 0.848 |
|  | Model2 | **0.354** | **0.009** | 0.166 | 0.234 | 0.061 | 0.663 | 0.133 | 0.344 |
| Maternal  Weight Gain | Model1 | 0.191 | 0.128 | **0.404** | **0.001** | -0.135 | 0.283 | -0.120 | 0.341 |
|  | Model2 | 0.181 | 0.194 | 0.175 | 0.210 | 0.186 | 0.182 | -0.040 | 0.776 |
| Cord Glucose | Model1 | **-0.355** | **0.008** | **-0.384** | **0.004** | 0.015 | 0.916 | -0.258 | 0.057 |
|  | Model2 | -0.156 | 0.284 | **-0.347** | **0.015** | -0.238 | 0.100 | **-0.446** | **0.001** |
| Cord C-peptide | Model1 | 0.178 | 0.193 | 0.210 | 0.124 | -0.127 | 0.356 | 0.095 | 0.488 |
|  | Model2 | 0.219 | 0.131 | -0.200 | 0.169 | -0.272 | 0.059 | -0.196 | 0.176 |
| Cord HDL-C | Model1 | -0.044 | 0.750 | -0.067 | 0.624 | **0.263** | **0.050** | 0.058 | 0.673 |
|  | Model2 | 0.014 | 0.921 | -0.095 | 0.510 | 0.053 | 0.715 | -0.125 | 0.387 |
| Cord LDL-C | Model1 | -0.049 | 0.721 | -0.192 | 0.157 | **0.401** | **0.002** | **0.274** | **0.041** |
|  | Model2 | -0.108 | 0.457 | -0.036 | 0.805 | -0.006 | 0.969 | -0.090 | 0.534 |
| Cord FFA | Model1 | -0.079 | 0.564 | -0.245 | 0.068 | -0.053 | 0.696 | -0.169 | 0.214 |
|  | Model2 | -0.083 | 0.568 | 0.015 | 0.917 | -0.104 | 0.474 | -0.175 | 0.224 |
| Cord Leptin | Model1 | 0.070 | 0.602 | -0.112 | 0.404 | -0.055 | 0.681 | 0.147 | 0.269 |
|  | Model2 | -0.102 | 0.474 | -0.145 | 0.306 | -0.186 | 0.187 | -0.077 | 0.587 |
| Cord Adiponectin | Model1 | 0.070 | 0.633 | 0.082 | 0.577 | -0.183 | 0.208 | 0.193 | 0.184 |
|  | Model2 | 0.068 | 0.663 | 0.268 | 0.083 | -0.061 | 0.697 | 0.240 | 0.121 |

Model 1 is non-adjusted.

Model 2 is adjusted for maternal age, parity, GDM and gestational age, and fetal sex.
